# A compactness measure of sustainable building forms

**DOI:** 10.1098/rsos.181265

**Published:** 2019-06-12

**Authors:** Bernardino D’Amico, Francesco Pomponi

**Affiliations:** REBEL (Resource Efficiency Built Environment Lab), Edinburgh Napier University, Edinburgh, UK

**Keywords:** sustainable buildings, material efficiency, compactness, building form, building design

## Abstract

Global population growth and urbanization necessitate countless more buildings in this century, causing an unprecedented increase in energy consumption, greenhouse gas emissions, waste generation and resource use. It is imperative to achieve maximal efficiency in buildings quickly. The building envelope is a key element to address environmental concerns, as it is responsible for thermal transfers to the outdoors, causing energy demand and carbon emissions. It also requires cladding, thus consuming a significant amount of finite resources. This paper investigates the relationship between surface area and indoor space to unravel the sustainability of building forms. Firstly, we demonstrate what the optimal form is. Secondly, as a single definite form is of little use in practice, we develop a scale-independent metric to measure the degree of optimality of building forms and show its practical use. This newly developed metric can significantly help in early design stages, by quantifying how much a building form deviates from optimality and identifying the domain of alternative geometries to bring us closer to it. This compactness measure also represents a theoretical basis for further research, to explore how optimality changes when additional parameters are factored in. It therefore contributes to both theory and practice to support global efforts towards sustainable built environments.

## 1. Background

Buildings are ubiquitous. The vast majority of human interactions, energy consumption and waste generation are related to—or take place in—buildings and cities [1]. Buildings and, consequently, cities are also the greatest single cause of anthropogenic greenhouse gas emissions (GHG) and the largest consumer of the Earth’s finite natural resources [2].

Buildings also account for over half of the global energy demand and more than a third of waste flows [3]. Due to unprecedented growth rates in human history for both global population and urbanization, countless more buildings are needed in the next decades. In fact, they can be counted: between now and 2030, cities will house an additional one billion people, which equates to building one new city of 1.5 million inhabitants (roughly the population of Manhattan) each week for the next 12 years [4].

Also, in spite of global efforts, International Energy Agency (IEA) projections suggest that carbon emissions are on track to double by 2050 [5]. Cities are already aware of their resource consumption and environmental impacts [6] but with such projections it has never been so imperative, timely and important to approach maximal efficiency in buildings to accelerate the transition to a sustainable built environment and avoid crossing that 2°C limit that would cause catastrophic and irreversible climate change [7].

In accommodating an additional one billion people while mitigating the negative impact on the natural environment at all levels, the need for an effective use of space in buildings is both evident and crucial. Arguably, each building is unique; and yet buildings can be surprisingly clustered around very few parameters. Two, in particular, suffice to provide a rough understanding of their forms and functions, namely:

— Floor Area^1^: for two-dimensional-driven spaces, that is when the lettable or walkable area is what defines the use and the value of a built asset, such as in dwellings and offices.
— Volume: for three-dimensional-driven spaces, in those cases where the volume is the key dimension of the building design, such as for theatres, museums, auditoria, lecture halls and so on.

In both cases, the building envelope is the key by which thermal transfer between indoor and outdoor space occurs, thus, it is directly responsible for the building's heating and cooling demands and related GHG [9,10].

### 1.1. The role of building forms

As referenced in the next section, minimizing the envelope surface area—for a given amount of enclosed volume (and/or floor area)—is a clear objective to approach maximal efficiency in buildings. In addition to mitigating thermal transfer, reducing the envelope surface can also help lower the embodied energy and environmental impacts linked to materials and products that are required to construct the envelope itself, especially considering that such building components are often characterized by energy- and carbon-intensive supply chains [11]. The relationship between envelope surface area (of a building or any three-dimensional object) and corresponding internal volume is indeed a geometric one. This ultimately suggests that some building forms are inherently more efficient than others, regardless of the material being used to realize them. There are, however, other elements related to a building envelope that do influence its energy consumption and the overall building sustainability—for instance, passive strategies for natural ventilation or the reduction of cooling loads—but they are outside the scope of this paper although would represent interesting areas for further expansion of the work presented here.

## 2. Previous works

Scholarly interest in building forms is not new [12], but the amount of existing literature does not seem to reflect the importance of the topic. Our understanding of, and explanation for, the lack of extensive literature on the topic is the overwhelming attention that has been sadly given to operational energy alone in the past decades [13]. This seems to be finally shifting, with publications and standards assessing the life cycle performance of buildings [14], and a greater social and political awareness of this important aspect, as demonstrated by the recent London Environment Strategy [15], the Buy Clean California Act [16] and local authorities that require reporting of embodied carbon [17]. From our literature review, two macro areas of research on building forms emerged.

The first one can be labelled as *classification-seeking*. This includes contributions aimed at a better understanding of the existing building stock. Such information is of vital importance, for instance, to run analyses aimed at identifying specific energy policies or to understand the effectiveness of refurbishment campaigns. This area also includes those efforts aimed at examining the evolution of the morphology of urban systems [18] as well as differences [19] and similarities [20] in urban forms and cities across the globe. Seminal contributions in the *classification-seeking* area come, for instance, from Steadman and colleagues [21,22] who surveyed the building stock of England and Wales to unravel its identifying features [23]. More recently, Julia *et al.* [24] developed a Bayesian-based method to define residential archetypes in urban building energy models in the US, while Hargreaves [25] developed an innovative method to convert average residential densities into sets of one-hectare three-dimensional tiles that aim to represent the dwelling stock in the UK. This latter method has since been applied to forecast how residential urban forms affect regional carbon emissions from retrofitting and decentralized energy supply [26], which is a perfect example of how a previously developed *classification-seeking* method is utilized to serve its purpose of analysing the effect of energy policies on the building stock.

The second macro area of research can be labelled as *form-seeking*, for it aims at identifying optimal or effective forms given a specific objective. This is the area of the seminal work of Leslie Martin [27] and Lionel March [12]—who posed the famous, and likely yet unanswered question *‘What building forms make the best use of land?’*—as well as the subsequent works that followed at the Martin Centre of the University of Cambridge. Some noteworthy examples are those of Steemers [28], investigating the building energy use in relation to urban built forms and density; Ratti *et al.* [29], exploring the effects of urban texture on building energy consumption through digital elevation models; and Ratti *et al.* [30], assessing the relationship between building forms and environmental performance through archetypes. Also falling in this area is the work done by Catalina *et al.* [31] who investigated the impact of building forms on energy consumption in the case of office buildings. They utilize a combination of a building shape factor (*L*_*b*_) and a building relative compactness indicator (*R*_*c*_) to optimize the shape and functional structure of energy-saving buildings. The relative compactness *R*_*c*_ has also been used by Ourghi *et al.* [32] in a simplified analysis to predict the impact of shape on annual energy use for office buildings. Similarly, Schlueter & Thesseling [33] developed a building information modelling (BIM) tool for energy and exergy performance assessment. These kind of studies, however, adopt the building form as the starting point rather than the final objective of a study. In terms of optimization, Jedrzejuk & Marks developed [34] and applied [35] a multi-criteria optimization approach that considers construction costs, seasonal demand of heating energy and pollution emitted by heat sources. For a review of optimization algorithms for building design, the reader is referred to Machairas *et al.* [36].

Outside of this line of enquiry, Hachem *et al.* [37,38] investigated housing unit designs in order to optimize urban settlements to exploit solar radiation. Similarly, Okeil [39] sought energy-efficient building forms by cutting solar profiles through a conventional block, thus developing what he called a Residential Solar Block (RSB) that maximizes the solar energy hitting the building’s façades. Conversely, Caruso *et al.* [40] aimed to explore geometric forms which minimize the direct solar radiation incident on the building’s envelope to reduce cooling loads. They found that for a given volume, *V*, the optimal form is represented by a family of ovoidal solutions, parametrized against the latitude of the building location. Jin & Jeong [41] developed an optimization method for free-form building shapes aimed at minimizing thermal loads through a genetic algorithm implemented in Rhinoceros.

Non-rectangular shapes have also been the subject of investigation in an attempt to minimize cooling loads [42] with the conclusion that a reduced envelope surface drives optimality in building forms aimed at lowering energy consumption. A result, this latter, in accordance with previous findings from Steadman *et al.* [9] who empirically demonstrated, for the entire non-domestic building stock of London, the existence of a strong correlation between exposed building surface area (walls plus roof) and gas/electricity use. Similar results have been also recently confirmed by Vartholomaios [43] in geographical contexts with warmer climates such as Greece, where he identified a synergy at different urban densities between compactness and passive solar design. Switching from solar radiation to air quality, Cheshmehzangi [44] investigated, through computational fluid dynamics (CFD) modelling, forms that yield maximum air quality in urban micro environments. Chau *et al.* [45] instead attempted to define optimal building heights in the context of Hong Kong by factoring in construction costs and property prices as well as the building height. They found that both costs and revenues increase with height, though the former rises faster than the latter, thus implying the existence of an optimal height from an economic point of view. In terms of buildings height, recent work by Helal *et al.* [46] questioned the premium-for-height method developed by Khan [47] by broadening the scope of the analysis and included additional driving factors such as embodied energy, water and carbon.

## 3. Aim and objective

The studies reviewed in the previous section highlight a wide range of objectives and parameters that can be considered in seeking optimal building forms. Yet, most results concur to the fact that reducing the external building surface, while increasing its internal volume, leads to lower energy consumption and a mitigation of related environmental impacts. Building on this simple but important observation, the main objective of this work has been to analytically derive a dimensionless factor to enable measurements of the extent to which variations of the geometric form affect the relationship between external surface area and the internal volume of building shapes. As such, the objective is aimed at providing a tool (suited to a broad range of stakeholders, such as designers, urban planner and policy makers) to help achieve a more sustainable use of resources and space. In this work, we refer, for sustainable development, to a passage from the Brundtland’s report (p. 9) which frames it as *‘a process of change in which the exploitation of resources, the direction of investments, the orientation of technological development, and institutional change are made consistent with future as well as present needs.’* [48]. From this definition, this article is primarily focused on two of the defined elements above. Firstly, it considers the exploitation of resources, both as materials used in the building envelope as well as fossil fuels and/or renewable energy technologies for the energy demand in the operational phase of buildings. Secondly, it aims to orient design therefore addressing the technological development of today’s and tomorrow’s built environment.

## 4. Analysis

A common approach adopted to quantify the relationship between the external surface area (*S*) of a building—or any other three-dimensional-solid—and its hosted internal volume (*V*) is to look at the ratio *S*/*V* [49,50], therefore preferring building forms scoring a low ratio as an indicator of high compactness. An important limitation, however, of the surface area-to-volume ratio is that such a parameter is not scale-independent, meaning that two building forms with the same exact shape (let say, a cube) but different size, will inevitably result in two different *S*/*V* ratios. More precisely, for a given geometric shape, the bigger the volume being considered, the smaller the ratio *S*/*V* becomes. Such a scale-dependency of *S*/*V* (also known as the square-cube law [51]) makes the surface area-to-volume ratio of little use for comparative shape analyses. When designing two-dimensional-driven building spaces, for instance, the total internal floor area (*A*_tot_) to be realized is usually known *a priori*, and so the building internal volume:

4.1 $$V=hA_{\mathrm{tot}},$$

(where h=inter-storey height) therefore, the designer’s task would be to compare alternative building shapes that match the given internal floor area (and hence volume) while reducing the required envelope surface area (*S*).

A new, dimensionless, factor is introduced here to consistently quantify the degree of compactness of building forms only as a function of their shape, thus regardless of their size (and volume). Applicability of such a compactness factor is limited to rectangular building forms. However, this limitation should not prevent the theoretical results presented here from finding a useful application in real design practice, given that the vast majority of buildings fall within this category [52].

### 4.1. Preliminaries

Given a generic three-dimensional building form, we consider *V* its internal volume, *S* its external surface area and *S*_min_ the minimum amount of external surface area required to enclose the given volume *V*.

A (dimensionless) compactness measure of the building form can be safely taken as the ratio *S*/*S*_min_. In such a case, the degree of compactness will be inversely proportional to the value of the ratio, with optimal (maximum) compactness achieved when *S*/*S*_min_ is at its minimum, i.e. when *S*/*S*_min_ = 1. The problem thus consists of defining a reference value for *S*_min_, or more precisely, to find a function *S*_min_(*V*) so that, for a given three-dimensional building shape with volume *V*, the corresponding ratio *S*/*S*_min_(*V*) can be computed.

Ideally, the sphere is the three-dimensional shape with minimum external surface area (*S*_sphere_) for a given volume *V* = *V*_sphere_, and therefore it makes perfect sense to derive *S*_min_(*V*) by looking at the relation between *S*_sphere_ and *V*_sphere_. In this case we would have:

4.2 $$S_{\mathrm{sphere}}=4\pi R^{2}\;\text{and}\;V_{\mathrm{sphere}}=\frac{4}{3}\pi R^{3},$$

with *R* being the sphere radius. Inserting the second equation (4.2) into the first equation (4.2), then rearranging, we obtain:

4.3 $$S_{\mathrm{sphere}}=4\pi \;\;{\;}^{3}\sqrt{{\left(\frac{3V_{\mathrm{sphere}}}{4\pi }\right)}^{2}}=\;{\;}^{3}\sqrt{36\pi V_{\mathrm{sphere}}^{2}}.$$

Remembering that *S*_sphere_ is the minimum surface to envelop a generic volume *V*, the degree of optimality of the generic surface *S* which wraps *V* can be captured by the following expression of compactness measure:

4.4 $$\frac{S}{S_{\min}}=\frac{S}{\;{\;}^{3}\sqrt{36\pi V^{2}}}.$$

Equation (4.4) is useful—and usable—to assess the degree of compactness of building forms regardless of their actual size. Yet, such a compactness ‘score’ would only tell us how ‘far’ from the (theoretically optimal) spherical shape a certain building form is. It would certainly be more useful to draw some general relationships between characteristic parameters defining the shape of buildings (e.g. in terms of footprint aspect ratio and/or slenderness ratio) and their effect on compactness.

### 4.2. Cuboid-like shapes

Neglecting spherical shapes, the geometric domain to derive a reference value for *S*_min_ can be limited to rectangular parallelepiped box shapes (also known as *cuboids*). In this case, the minimum surface area—excluding the part of the surface in contact with the ground—required to enclose a given volume *V* is:

4.5 $$S_{\min}(V)=3\;\;{\;}^{3}\sqrt{4V^{2}}.$$

**Proof.**

**Figure 1.**
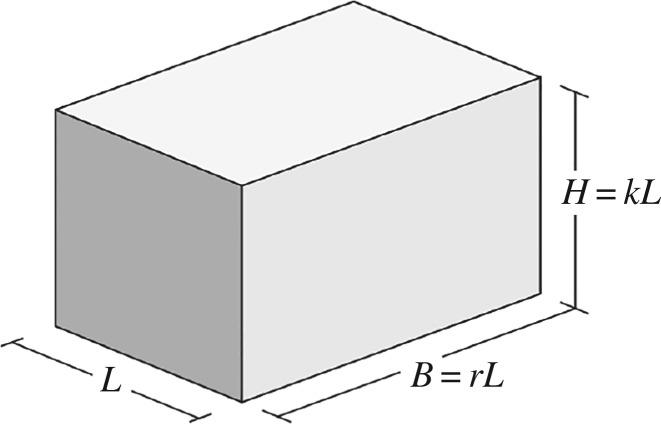
Cuboid-like building shape, parametrized in terms of footprint aspect ratio (*r*) and slenderness aspect ratio (*k*).

Let us consider a generic cuboid having width = *L*, depth = *B* and height = *H*, thus its shape can be expressed in terms of footprint aspect ratio (*r*) and slenderness aspect ratio (*k*) as follows (see figure 1):

4.6 B=rLandH=kL,

which yields the following expressions for *S* and *V*:

4.7 $$S=rL^{2}+2rLH+2LH\;\text{and}\;V=rL^{2}H.$$

Note: the part of the external surface in contact with the ground has not been considered in the first equation (4.7). In order to demonstrate equation (4.5), we are first going to determine for what value of footprint aspect ratio (*r*) the surface area (*S*) is minimized. From the second equation (4.7), we have:

4.8 $$L=\sqrt{\frac{V}{rH}}\;,$$

which inserted into the first equation (4.7) enables us to express the surface area as a function of volume, height and footprint aspect ratio:

4.9 $$S(V,H,r)=\frac{V}{H}+2H\sqrt{\frac{V}{H}}r^{1/2}+2H\sqrt{\frac{V}{H}}\frac{r^{1/2}}{r}.$$

For a given *V* and *H*, the value of *r* such that *S* is minimized can be found by setting the partial derivative of *S* with respect to *r* as equal to zero:

4.10 $$r⟶\frac{\partial S}{\partial r}=0,$$

where:

4.11 $$\frac{\partial S}{\partial r}=H\sqrt{\frac{V}{H}}r^{-1/2}+\frac{1}{r^{2}}\left(H\sqrt{\frac{V}{H}}r^{-1/2}r-2H\sqrt{\frac{V}{H}}r^{1/2}\right)=0,$$

which rearranged becomes:

4.12 $$\frac{H}{\sqrt{r}}\sqrt{\frac{V}{H}}\left(1+\frac{1}{r}-\frac{2}{r}\right)=0.$$

Multiplying both sides of equation (4.12) by $\sqrt{rH}/(H\sqrt{V})$ gives:

4.13 $$\frac{1}{r}-1=0,$$

which has solution *r* = 1, meaning that the surface area is minimized when the building footprint is a perfect square. Indeed, this holds true for any volume and, most important, for any given height. Therefore, by setting *r* = 1 in equation (4.9):

4.14 $$S(V,H)=\frac{V}{H}+4\sqrt{V}H^{1/2},$$

the value of *H* such that the external surface is minimized can be found by setting the partial derivative of *S*(*V*, *H*) with respect to *H* as equal to zero:

4.15 $$H⟶\frac{\partial S}{\partial H}=0,$$

where:

4.16 $$\frac{\partial S}{\partial H}=-\frac{V}{H^{2}}+\frac{2\sqrt{H}}{H^{1/2}}=\frac{-V+2\sqrt{V}H^{3/2}}{H^{2}}=0,$$

which rearranged becomes:

4.17 $$2\sqrt{V}\sqrt{H}H=V,$$

providing the following value for *H*:

4.18 $$H=\;\;{\;}^{3}\sqrt{\frac{V}{4}}.$$

The height *H* can now be expressed as a function of the building width *L*. Remembering that the optimal footprint aspect ratio is equal to 1, inserting the second equation (4.7) into equation (4.18) we have:

4.19 $$H^{3}=\frac{V}{4}=\frac{L^{2}H}{4}\Rightarrow H=\frac{L}{2}.$$

Therefore, for any given volume *V*, the minimum surface area *S*_min_ for a cuboid is obtained when *r* = 1 and *H* = *L*/2, which corresponds to the shape of a semi-cube. For such conditions equation (4.7) becomes:

4.20 $$S=3L^{2}\;\text{and}\;V=\frac{L^{3}}{2},$$

and therefore the expression of minimum surface area (4.5) can be obtained by inserting the second equation (4.20) into the first equation (4.20). □

A compactness measure alternative to equation (4.4) can now be derived from equation (4.5) as follows:

4.21 $$\frac{S}{S_{\min}}=\frac{S}{12V}\;\;{\;}^{3}\sqrt{16V}.$$

### 4.3. Parametric study

In order to analyse how the footprint aspect ratio (*r*) and slenderness ratio (*k*) affect the compactness (*S*/*S*_min_) of cuboid-like building shapes, we first need to express the external surface area *S* as a function of *r* and *k*. Inserting the second equation (4.6) into equation (4.9) we have:

4.22 $$S(V,r,k)=\frac{V}{kL}+\frac{2Lk}{\sqrt{k}}\sqrt{\frac{Vr}{L}}\left(1+\frac{1}{r}\right)\;\text{where}\;L=\;\;{\;}^{3}\sqrt{\frac{V}{rk}},$$

which rearranged in a more compact form becomes:

4.23 $$S(V,r,k)=\;\;{\;}^{3}\sqrt{V^{2}}\left[\;\;{\;}^{3}\sqrt{\frac{r}{k^{2}}}+2\;\;{\;}^{3}\sqrt{rk}\left(1+\frac{1}{r}\right)\right],$$

and divided by *S*_min_, as from equation (4.5), leads to the sought relationship between compactness and building shape parameters (*r* and *k*):

4.24 $$\frac{S}{S_{\min}}=\frac{1}{3\;\;\;{\;}^{3}\sqrt{4}}\left(\frac{2rk+r+2k}{\;\;\;{\;}^{3}\sqrt{r^{2}k^{2}}}\right).$$

It is worth noting that the volumetric term (*V*) has disappeared from the right side of equation (4.24), since the compactness metric *S*/*S*_min_ is indeed scale-independent. We believe that equation (4.24) has useful implications as it enables the measurement of the degree of compactness of a building only with regard to its shape—parametrized in terms of footprint aspect ratio and slenderness aspect ratio.

A further development of the theory, described so far for cuboid-like building shapes, concerns the possibility to express the ratio *S*/*V* of a generic cuboid as a product between two terms:

— A scale-dependent term, *β* (that we call here *size factor*) which provides the minimum *S*/*V* ratio achievable for any given building volume *V*.
— A scale-independent factor, *γ* (called here *shape factor*) which accounts for any additional increase of *S*/*V* as a result of variations of the (footprint and slenderness) aspect ratios.

In mathematical terms:

4.25 $$\frac{S}{V}=\frac{S_{\min}}{V}\frac{S}{S_{\min}}=\beta (V)\gamma (r,k),$$

where *β*(*V*) is derived by dividing the right-side term in equation (4.5) by *V*, whereas *γ*(*r*, *k*) is that already given in equation (4.24). Therefore:

4.26 $$\beta (V)=3\;\;{\;}^{3}\sqrt{\frac{4}{V}}\;\text{and}\;\gamma (r,k)=\frac{1}{3\;\;{\;}^{3}\sqrt{4}}\left(\frac{2rk+r+2k}{\;\;{\;}^{3}\sqrt{r^{2}k^{2}}}\right).$$

## 5. Results and discussion

**Figure 2.**
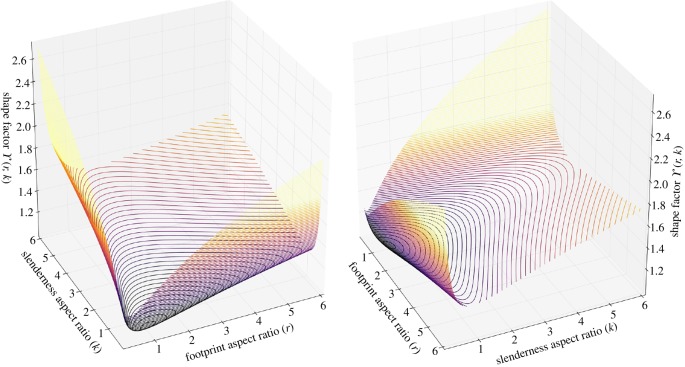
Two views of three-dimensional plot of the *shape factor* *γ* as function of footprint aspect ratio and slenderness aspect ratio. See second equation (4.26).

A plot of the *shape factor* as a function of footprint and slenderness aspect ratios has been numerically generated and shown in figure 2. The surface function *γ*(*r*, *k*) reaches its global minimum, *γ* = 1, when *r* = 1 and *k* = 0.5—see equations (4.13) and (4.19)—i.e. when the cuboid takes the shape of a semi-cube.

**Figure 3.**
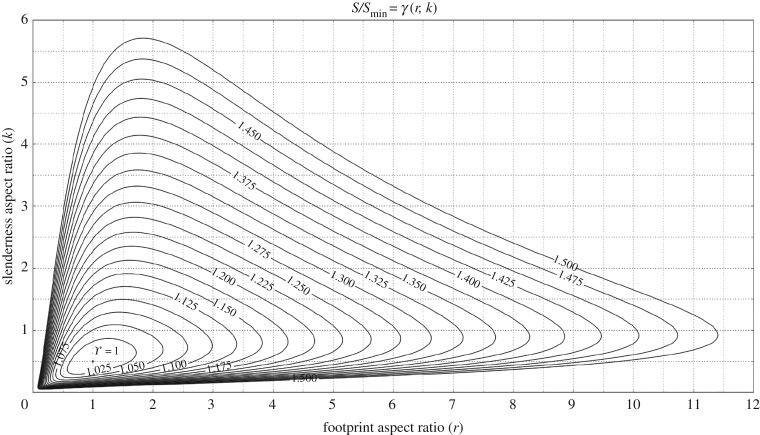
Two-dimensional plot in the *r*–*k* plane of the *shape factor* *γ*. Numerical values labelled on the level curves indicate the corresponding *γ* value at that curve.

The same function *γ* is shown in figure 3, mapped in the *r*–*k* plane, where the numerical values labelled on the level curves indicate the corresponding *γ* value at that curve. Each level curve defines the set of pairs (*r*, *k*)—that is to say, the set of building shapes—for which a certain compactness (*shape factor*) is being achieved, therefore making such a graphical representation practically useful for building design purposes.

A valuable insight provided by figure 3 in this regard is that, in the vicinity of the semi-cube solution (i.e. for *γ* ≈ >1), the *shape factor* *γ* increases at a small rate, whereas a higher rate of increase is found for *γ* as we move away from the optimum. For example, a shape with footprint aspect ratio *r* = 2 and slenderness ratio *k* = 1 (i.e. twice the optimal values *r* = 1; *k* = 0.5) has a *shape factor* *γ* ≈ 1.05 (i.e. only ≈5% higher than the optimal). On the other hand, a shape with footprint aspect ratio *r* = 4 and slenderness ratio *k* = 2 (i.e. four times the respective optimal values) has a *shape factor* *γ* ≈ 1.26 (i.e. about 26% higher than the optimal). A practical implication of this observation is that there exists a wide range of geometrically different shapes to choose from, which are only slightly less efficient than the theoretically optimal semi-cube shape.

### 5.1. Reciprocal footprint aspect ratios

**Figure 4.**
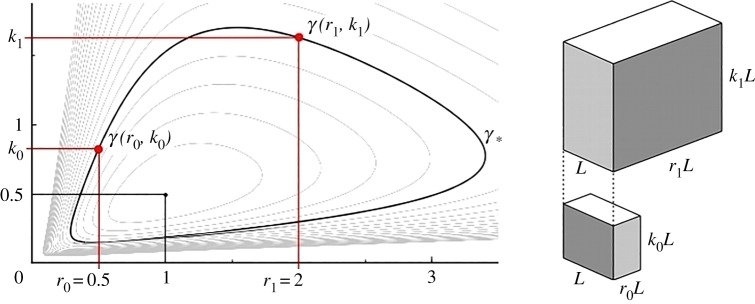
For a given *shape factor* =γ_*_ there exist two pairs of variables (*r*_0_, *k*_0_) and (*r*_1_, *k*_1_) representing the same shape—i.e. the first cuboid is obtained by uniformly scaling the second one and rotating it by 90°.

It should be noted that for a given *shape factor* (level curve) there always exist two distinct pairs of variables (*r*, *k*) representing exactly the same shape—or more precisely: one cuboid can be obtained by applying a 90° rotation and a (uniform) scaling to the other cuboid (figure 4).

In particular, given a *shape factor* γ_*_, there exist two pairs of values (*r*_0_, *k*_0_) and (*r*_1_, *k*_1_):

5.1 $$\gamma_{*}=\gamma (r_{0},k_{0})=\gamma (r_{1},k_{1}),$$

such that the footprint aspect ratios *r*_0_ and *r*_1_ are *reciprocal*:

5.2 $$r_{1}=\frac{1}{r_{0}}.$$

To avoid considering the same building shape twice when looking at the graph *γ*(*r*, *k*) it is sufficient to consider only the part of level curves for *r* ≥ 1, or vice-versa, only the level curves for *r* ≤ 1.

### 5.2. Equivalent slenderness aspect ratios

Restricting the domain of the *γ*(*r*, *k*) function to *r* ≥ 1, it can be shown that given a value of footprint aspect ratio r_*_, there exist two different values of slenderness aspect ratio *k* (e.g. *k*_0_ and *k*_1_) such that:

5.3 $$\gamma (r_{*},k_{0})=\gamma (r_{*},k_{1}).$$

**Figure 5.**
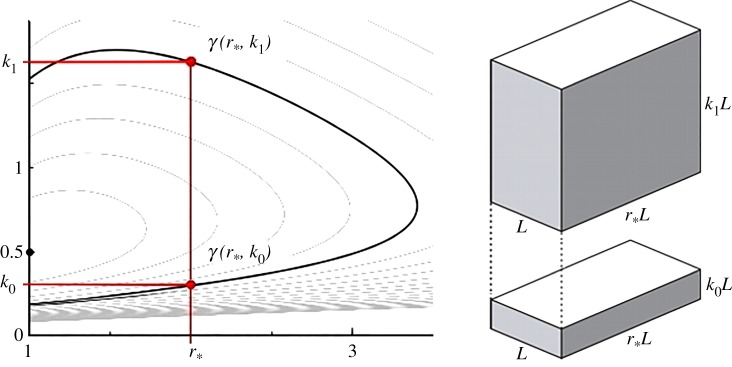
Two cuboid shapes having shame *shape factor *γ** and same footprint aspect ratio, r_*_, but different slenderness aspect ratio.

Meaning that for a given *shape factor* there exist two cuboids whose shapes only differ in terms of slenderness. Such an equivalence between slenderness aspect ratios is graphically illustrated in figure 5.

### 5.3. Surface-to-volume ratio

**Figure 6.**
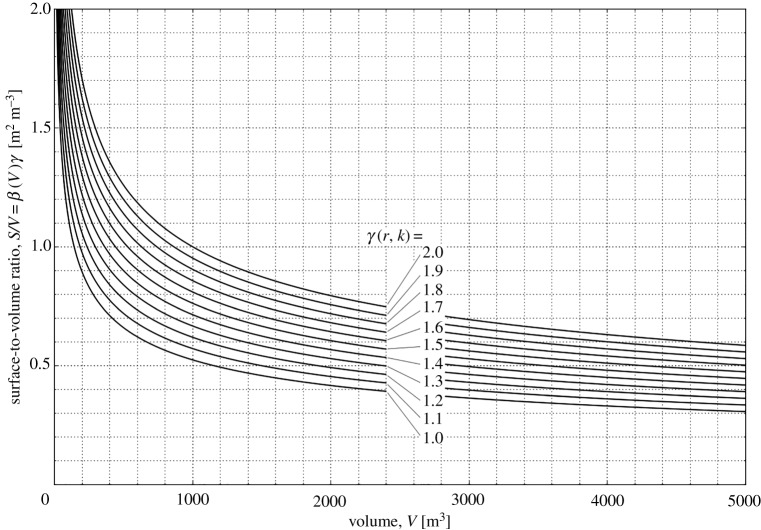
Surface-to-volume ratio as a function of the building volume and *shape factor*.

As discussed in §§4–4.3, the surface-to-volume ratio, *S*/*V*, of cuboid-like building shapes can be decomposed in two separate terms: a scale-dependent *size factor*, *β* (measurable in m^2^ m^−3^), and a dimensionless *shape factor*, *γ*. A graphical representation of the function *S*/*V* = *β*(*V*)*γ* is provided in figure 6, based on equation (4.26), for *shape factor* values ranging from 1 to 2. The plot clearly shows that the surface-to-volume ratio decreases as the building volume increases.

It can be seen that the *shape factor* *γ* has greater influence over the scale factor for smaller volumes, due to the slope of the *S*/*V* function decreasing as the volume increases. However, in relative terms, things are different, since for a volume of 200 m^3^, the minimum *S*/*V* ratio (i.e. for *γ* = 1) is $\approx 0.9\;{\text{m}}^{2}\;{\text{m}}^{-3}$ which increases by ≈88% for a *shape factor* *γ* = 2. If we consider the 5000 m^3^ instead, a *S*/*V* ratio of circa 0.3 m^2^ m^−3^ is found for *γ* = 1, which increases by ≈100% when a *shape factor* *γ* = 2 is assumed.

## 6. Case studies

**Figure 7.**
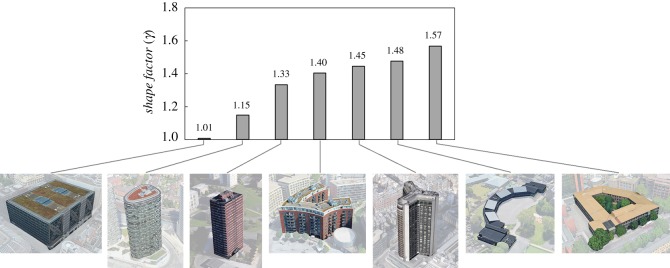
Compactness of building forms measured in terms of *shape factor* *γ* = *S*/*S*_min_ as from equation (4.21). Images source: Google Earth [53].

To demonstrate the practical usefulness of the metric developed, we have utilized a number of case studies. Following the same order in which the theoretical and mathematical findings have been presented, we start by focusing on buildings with any given shape and show how to use the *shape factor* *γ*. This is graphically shown in figure 7. Seven buildings in London and Edinburgh have been chosen that would constitute a good mix of different shapes and sizes. Details of the location for each building are provided in the figure, and basic geometric characteristics have been obtained from GIS data and then post-processed to calculate surfaces and volumes.

The building on the far left-hand side of the figure was intentionally chosen to (1) demonstrate the existence of buildings with highly optimal forms and to (2) serve as a reference benchmark for other building shapes. Moving from left to right, it can be seen how the shape factor increases leading to forms with values that are very far from optimality. It is not hard to imagine that during the initial design stages of these six buildings—which are incidentally all placed in areas that would have allowed slightly different footprints—the information on the degree of the (un)optimality of the shape could have driven the design team towards better forms while providing the same function (i.e. floor area or internal volume).

**Figure 8.**
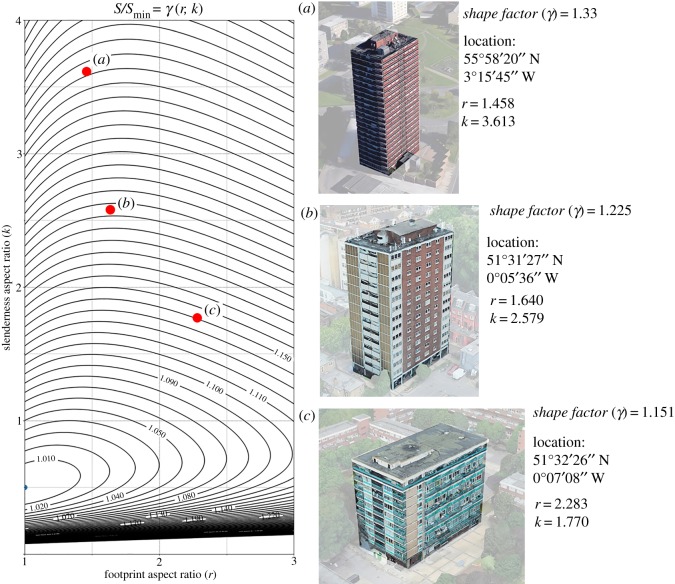
*Shape factor* *γ*(*r*, *k*) of domestic building forms as from equation (4.24). Images source: Google Earth [53].

We want to further demonstrate the usefulness of our metric in the case of both domestic and non-domestic buildings that fall within the cuboids category. These case studies have also been retrieved and measured through GIS data. Figure 8 shows three examples of domestic buildings (blocks of flats) with varying degrees of a *shape factor*. Being cuboids, it is now possible to precisely place them in the *r*–*k* plane (figure 3). Case study (c) in figure 8 has a much better form than (a) and (b). Similarly to the case of the buildings shown in figure 7, knowing the exact *γ* at the design stage would have allowed us to drive design efforts towards a better form, closer to optimality. In this case, however, the exact information on where each building sits in the *r*–*k* plane also enables us to understand which ‘interventions’ would be beneficial. For the case study (c), for instance, a reduction in the value of *r* would have moved the building onto a curve level characterized by a lower *γ* value. It is worth noting that this is not true for the case studies (a) and (b) as they belong to the region of the *r*–*k* plane where level curves are increasing towards their peak of *k*. For buildings (a) and (b) it would have been necessary to reduce the slenderness ratio (*k*) to move them on to the adjacent level curve characterized by a lower *γ* value.

**Figure 9.**
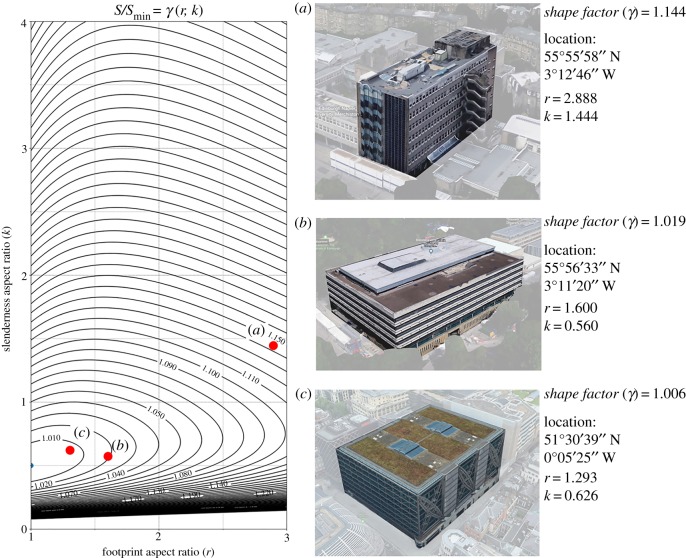
*Shape factor* *γ*(*r*, *k*) of non-domestic building forms as from equation (4.24). Images source: Google Earth [53].

A similar application of our metric in the case of non-domestic buildings is shown in figure 9. Case study (c) is the very optimal building already shown in figure 8, whereas (a) and (b) are two non-domestic buildings located in Edinburgh. Contrary to what was shown in figure 8, in this specific case, a reduction of the footprint aspect ratio (*r*) would have been beneficial to both buildings. However, a reduction of *k* would have been beneficial to building (a) only since building (b) is in an area of the *r*–*k* plane where a reduction of *k* would have moved the building onto the adjacent level curve which is characterized by a higher *γ* value.

We have therefore shown that, with simple information and basic calculations which are instantaneous in contemporary digital design tools, it is possible to grasp a quick understanding of the optimality of a built form. This metric can be useful for buildings of any given shape (figure 7) as well as cuboids (figures 8 and 9). In the latter case, our metric provides much richer information since it allows us to place the building on an exact point of the *r*–*k* plane, making the variations of the design of the building form that would be beneficial instantly recognisable.

## 7. Implications for research and practice

While the aim of this work has been mainly theoretical, some implications for both research and practice have arisen. On the research side, it appears that the current focus on greater operational efficiency of buildings as well as the materials employed neglects the importance of the building shape itself for the overall whole-life impact of buildings. Sustainability-oriented research could therefore move to a more holistic approach where the level of analysis goes from single materials, components or life cycle stages to a comprehensive assessment of the building as a whole.

Practically, the scale-independent metric developed here can provide support to real-world building design as we have shown in the case studies section. For instance, many (parametric modelling based) software tools can now calculate surfaces and volumes of any arbitrarily complex shape. Yet, this information is not then used to understand how far from a theoretical optimality that particular shape is. Our proposed (scale-independent) compactness measure can be easily programmed into such software tools, to instantly provide information on the optimality of the shape at hand and suggest potential directions (e.g. increased slenderness or increased footprint) in order to reduce the whole-life energy/carbon/resource impacts. We have also shown the possibility of utilizing our metric for non-cuboidal shapes, again as a rapid ‘feeler’ for the goodness of any building shape. We want to stress that our intention is not to prescribe design or reduce freedom and creativity, but rather to enable more awareness of choices made at the design stage and increase consciousness of their implications.

## 8. Conclusion

In this work, we have investigated the relationships between the envelope surface and the indoor spaces in buildings to establish which forms embed inherent sustainability. In doing so, we have considered the envelope surface (walls plus roof) as the key element to define buildings’ shapes, for it is the main cause of thermal transfer with the outdoors—thus creating energy demand and carbon emissions—and it also requires a substantial amount of materials produced form finite natural resources, that are often replaced multiple times throughout a building’s service life.

Under the realistic assumption that most buildings are rectangular, we have analytically demonstrated which is the optimal form that minimizes the envelope surface, that is a semi-cube. Given that a single form is of little use in practice, we have used that as a benchmark to develop a scale-independent metric (*shape factor*) to measure the compactness of building forms as a function of their shape—parametrized in terms of footprint and slenderness aspect ratios.

In practice, this materializes into a useful tool to quantify, and thus understand, how much a given building form deviates from a theoretical optimality, regardless of its size. Additionally, our graphic tool could also point towards alternative forms that are instead closer to optimality.

We have demonstrated that the surface-to-volume ratio of any building can be expressed by a product of two distinct parameters: a scale-dependent term (what we called a *size factor*) and a scale-independent term (the *shape factor*).

While this article is chiefly theoretical in nature, its findings can support the design process of buildings, particularly at early stages when the room for improvement is at its maximum with little or no extra cost. By being able to quantitatively measure the degree of optimality of building forms, it is hoped that future designs will take this into account and contribute towards a greater sustainability of our built environments. We have demonstrated the practical usefulness of our metric in a number of case studies of real buildings.

The findings from this paper also constitute a valuable theoretical basis for further research. We have limited our investigation to the envelope surface, for it is a recognized key element that drives energy consumption and carbon emissions in buildings. However, there are certainly other factors that can play an additional role in the overall performance of a building. These could be taken into account in future works, to understand how optimality changes when more criteria are factored in.

## Footnotes

1 In this manuscript, floor area is used as a shorthand for the Gross Internal Floor Area, as defined by the Royal Institution of Chartered Surveyors [8]. Open Access publication was made possible thanks to FP's Vice Chancellor's Fellowship at Edinburgh Napier University.

## Data accessibility

This article has no additional data.
